# Identification and fixing bottlenecks of a food manufacturing system using a simulation approach

**DOI:** 10.1038/s41598-023-39025-5

**Published:** 2023-07-21

**Authors:** Seyed Mahdi Javadi, Seyed Jafar Sadjadi, Ahmad Makui

**Affiliations:** Iran University of Science and Technology, Tehran, Iran

**Keywords:** Mechanical engineering, Software

## Abstract

In manufacturing systems, simulation modeling plays an important role in creating some changes instead of working on real systems. Manipulation in a real system is more costly than manipulation in a simulated model. In this research, we tried to use a simulation approach to recognize and minimize bottlenecks of a production line, which will decrease costs and improve productivity. To achieve our objectives, we chose a case and analyzed its production line. By using our case study strategy, we tried to collect our data and adapt a conceptual model of production processes drawing on an operation process chart (OPC). After that, we created a simulation model of the production processes by using the popular Arena simulation software 13.5. By running the Arena model, some bottlenecks were found. Ultimately, we proposed some solutions to obviate bottlenecks and reduce the total costs of production.

## Introduction

**Figure 1:**
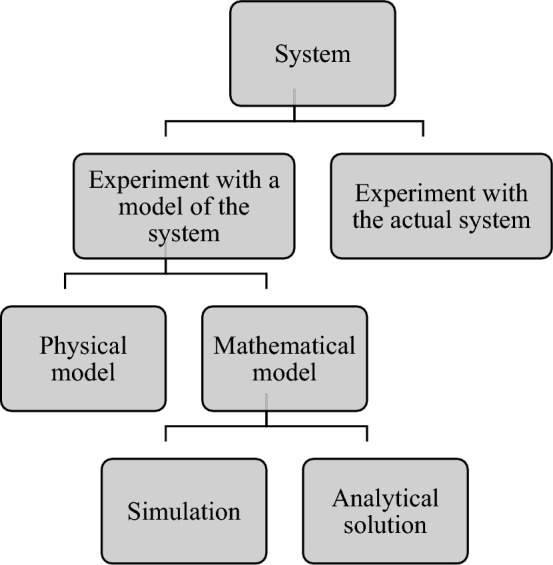
Ways to study a system.

A system is an integrated composite of people, products, and processes that provide a capability to satisfy a stated need or objective. A system includes facilities or processes that can be actual or planned, such as manufacturing facilities, banks, computer networks, business processes, chemical plants, supermarkets, airports, hospital facilities, and transportation. The system can be studied in different ways, as illustrated in Fig. 1.

Using a simulation model instead of a real system has several advantages. The most important advantage is that any failures occurred from changes applied in simulated model will not affect the profitability of the industry, because a simulation environment is safe for experimenting. While any failures occurred from changes applied in real model can cause exorbitant costs. Thus, by creating a simulated model of a real model, we are able to check out occurring events by manipulating certain changes or making new conditions that are not easy to control. Another advantage is that simulation helps companies to study various production scenarios that they cannot examine in real systems^1^.

Simulation is one of the most powerful analysis tools available to those in charge of the design, analysis, and operation of complicated processes or systems. In an increasingly competitive business world, simulation has become a very powerful tool for the planning, design, and control of systems. It is currently considered an indispensable problem-solving methodology for engineers, designers, and managers. In particular, simulation can play an important role in the analysis of manufacturing systems. As populous publication shows, simulation applications have been carried out on plant layout and utilization, testing control rules and inventory systems^2^. Some of the applications of the simulation approach are discussed in the following.

## Literature review

A study on simulation modeling in a hospital was performed using the Dynametrics software package to improve efficiency. The processes of ferrying patients to and from wards in a hospital were simulated, and some solutions were recommended to present the benefits available to health care managers through computer simulation^3^. Discrete event simulation is an effective tool that can be used to find operative solutions to manage an office. Simulation represents how efficiency can be improved by managing available resources and times taken to accomplish tasks^4^.

An application of simulation modeling in a hardwood sawmill was presented to justify the substitution of the trimmer in the hardwood sawmill, specify the effects of machine substitution on the hardwood sawmill, and denote the impacts of the availability of a forklift in the green chain. The study was completed in two phases. In the first phase, the sawmill operations and effects of machine substitution were determined. In the second phase, the simulation model was developed to include green chains by using Arena^5^. The deficiencies in current techniques in the hierarchical simulation of manufacturing systems have been presented. The first deficiency is the lack of information in order to do the process of changing levels of modeling details. The second is the deficiency of software support to help the user^2^.

Two major interests have been applied for the Arena software package. First, how to store a simulation model in an appropriate database; Second, how to retrieve a model from a database that is the most similar model to the specified model. The second interest has been investigated by a numerical approach in which a ranking number is assigned to the selected models such that the low number illustrates the best fit of the retrieved Arena model from the database to the specified model^6^. A novel rule-based method to extend flexible manufacturing system simulator software, ARENA software, has been provided by applying an object-oriented approach. The recommended procedure involved the use of a unified modeling language (UML) to reply to the requirements of the simulation developer by determining the goals of the model extension and by guiding the modeler in the running of the process^7^.

A research project has been carried out to develop an integrated methodology for planning and implementing the best manufacturing practice by tools that include SIMETRICS, Aris Tool Set, and ARENA. The modeling and simulation environment is one of the research project components. The methodology includes five phases: (1) the enterprise assessing drawing on performance indicators; (2) simulation modeling of the AS-IS core process; (3) choosing the best manufacturing practice; (4) simulation modeling of the TO-BE core process; and (5) assessing manufacturing practice implementation based on their advantages and effects^8^. Simulation is one of the most flexible procedures in the manufacturing systems design and operation fields. Overview research has been carried out to classify simulation problems into three categories: (1) manufacturing system design; (2) manufacturing system operation; and (3) simulation language/package development^9^.

Using optimization tools for a simulation model is one of the most important issues to search for an optimal strategy. Integration of ARENA and CPLEX, an optimization tool, has been applied to solve mixed integer programming (MIP) in a lower run time for large-scale problems^10^. A simulation approach has been applied to model an inspection plan for a multistage manufacturing system by the Arena simulation software package. Moreover, an optimal solution has been investigated to minimize the total inspection related to costs subject to a predefined average current quality by utilizing the OptQuest tool, an optimization component of Arena^11^. Discrete event simulation modeling is a strong tool in radiation therapy (RT). A simulation model of RT planning has been presented through Arena to recommend enhancements that probably reduce the planning time and finally decrease the total waiting times^12^. A simulation model of reverse logistics networks for gathering end-of-life products such as acid batteries has been provided by Arena 11.0 to analyze future network performance and to determine the unintelligible relationship between the parties involved in business^13^. A standard equation-based simulation model of the Russian macroeconomy in an agent-based setup remodeled to investigate the effect of antimonopoly legislation on long-term dynamic behavior^14^. Bhushan (2017) developed a sustainable humanitarian supply chain model consisting of different delivery subsystems for community development and used it for simulation analysis of Indian tribal communities^15^. Yatimi and Aroudam (2018) proposed a maximum power point tracking technique (MPPT) with variable weather conditions. They analyzed the output behavior and the performance of the system by MATLAB/Simulink simulation software to represent the validity of the method^16^.

Simulation-based applications in various manufacturing and technological fields and recent studies about the future of computing services such as the Internet cloud and the Internet of things were investigated and discussed^17^. A hybrid backwards-scheduling method (HBS) for discrete manufacturing systems was presented. This method works with a set of conversion relations to transmute a limited capacity forwards scheduling technique to their backward counterparts^18^. Three production scenarios with using simulation models were investigated by Tecnomatix Plant Simulation^19^. A hybrid approach of analytic and simulation methods were presented to solve production–distribution problems in supply chains^20^. An ARENA simulation model was proposed to minimize holding cost and set-up cost^21^. A methodology has been proposed for testing project management simulation games^22^.

Simulation modeling is a common method to analyze complicated systems. Simulation modeling of industrial systems includes manufacturing systems (such as production lines, inventory systems, workshops), supply chains, communicational and computer systems (for example, customer-service provider systems, communicational networks, etc.) and transportation systems (like airports and harbors). In fact, this method presents a simple form of the system under study. After that, this method will be developed to examine the system by determining objectives such as improvement of system design, profit-cost analysis, sensitivity to design parameters, and so on^23^. The motivation of this research is to identify the bottlenecks of a continuous production line that is not as easy as a discrete event production line. Novelty, we studied a continuous manufacturing system in the oil industry, and we could make a discrete event simulation model by considering entrance entities in quintuplet batch size to identify and fix bottlenecks of the production line.

In this paper, we selected an oil company in Iran, which produces liquid, solid, soybean, and sunflower oil, to identify obstacles to production. We will try to present some solutions to eliminate the barriers so that the quantity of production and productivity are maximized, and the total costs of production that have been formulated in Javadi and Mehrjerd (2016)^24^ include materials consumed cost, direct labor cost, and overhead cost, are minimized. If other companies use our findings, this simultaneous optimization will lead to enhanced domestic oil production and reduced imports of oil from other countries. To achieve our goals, we adapt the conceptual model of oil production of our case in the next section.

## Problem classification

### Simulation model categories

Drawing on a literature review, we can classify simulation models into three general categories^23^. These models include:

1. Physical models: a designer’s small preliminary model or sketch i.e. maquette. Flight simulators that are used to learn pilots and building replica model are two samples of physical models.
2. Analytical or mathematical models: a set of equations and relations among mathematical variables on how a system operates. Linear optimization problems, especially transportation problems, are an example of mathematical models. If the system is simple, it will recommend that mathematical analysis be carried out by using mathematical tools such as queue theory and mathematical programming to obtain the exact answer and establish a fairly complete understanding of the system. However, the systems are very complicated in the real world, and we can rarely show that they are validated by analytical models. When we use analytical models, we should be aware that considering the assumptions that make the model too simple can void the accuracy of the analytical model. A complicated system often needs a complicated model, and analytical methods are not used to model these systems. Because of inefficiency in the analytical model, we should use computer simulation as a strong and valid tool for modeling.
3. Computer models: These models are used to study a variety of ranges of real systems. In this method, studying the models is numerical and is done by software that is designed to simulate the operations, behavior and characteristics of the system. The real strength of simulation is its capability to study and check complicated, dynamic and stochastic systems such that we cannot easily obtain correct and fine answers by using analytical methods.

In the next section, we will peruse the operation process chart (OPC) of the production line. The conceptual model of the processes will be extracted. After that, we will implement the computer simulation model of our case company.

### Conceptual model

To analyze the case, we should adapt the conceptual model of the production line. First, the processes and operations that are done on raw materials, work in process, and goods should be checked.

A summary of the conceptual model to implement the Arena model states that at the first element, crude oil is entered to the production line by Tanker in a quintuplet batch size, and tankers stand on the bascule. Then, oil samples will be tested by the Operators of the Laboratory. If oil was safe, it will be vacated in oil Storages. Otherwise, oil will be returned. In the next stage, safe oil will enter the PX60 Separator, a small percentage will depart to the Soup Sewage, the rest of the oil will be directed to the SRG Separator (First Washing), some oil will enter the Soup Sewage, some percentage will be directed to the First of Neutral Line (starting of the PX60 Separator), and the rest will depart to the SSG Separator (Second Washing). After this stage, a small percentage of oil enters the First Neutral Line and the rest enters the Dryer. After the Dryer section, oil will be tested and will depart to the Bleaching (or Discoloring) section and will be tested again and will be strewed into Storages. After this stage, the production line breaks down into two lines: the liquid oil line and solid oil line. In the liquid oil production line, the oil will be moved from storage to the Odorlessing section. After that, some percentage will depart to fatty acid storage, and the rest will move to the laboratory.

In the next stage, the oil will be transferred to packaging storage, and after testing in the laboratory, it will be entered in a Serak machine to fill the bottles. At the end, in the packaging section, the bottles will be set in boxes and will be conveyed to solid and liquid storage by conveyers and will be maintained until distribution in the market. In the solid oil production line, the oil will enter the hydrogenation section, and after testing in the laboratory, it will depart to the filtering section and will test again and will move to the post bleach section. In the next stage, the oil will be vacated in the storage areas and then depart to the Odorlessing section. After this section, some percentage of the oil will move to fatty acid storage, and the rest will move to the laboratory. After testing, the oil will be transferred to the packaging section to set it to tins. Then, tins will be transported to cold storage by Liftruck. At the end, they will move to liquid and solid storage to distribute in the market.

**Figure 2:**
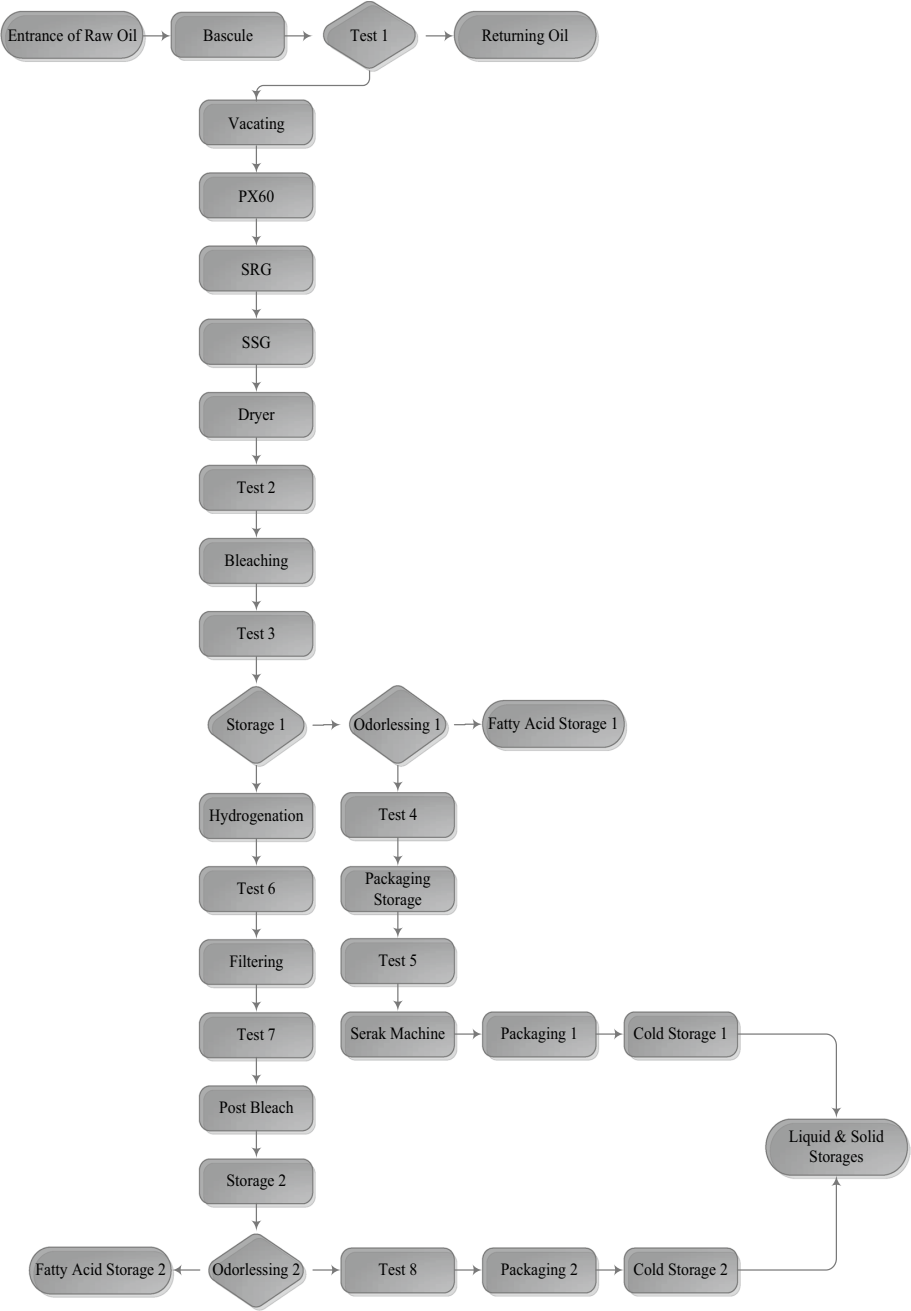
The conceptual model.

The conceptual model of the production line was extracted in the shape of a flowchart by OPC analysis and interviewing the production manager. This model was implemented when raw oil entered the production line until the distribution time of the goods to the market. The conceptual model is shown in Fig. 2, provided by Microsoft Visio 2010.

## Simulation approach

In the previous sections, we told that one of the most important reasons for using the simulation method in modeling our problem is the capability of examination and making some changes in the model with the lowest cost when creating changes in a real system has surplus costs and sometimes the changes are impossible.

There are different simulation problems as follows^1^:

- Static vs. dynamic problem: answering the question “does time play role in the model or not?” is a clear difference between static and dynamic problems.
- Continuous-change vs. discrete-change problem: statues changing based on continuous or discrete points in time are the scale of partitioning.
- Deterministic vs. stochastic problem: random or nonrandom inputs of the model will classify the problem to definite or stochastic problems.

According to the above explanation, because in our problem time plays an important role, and changing in system occurs in discrete points of the time, and inputs of the model have probably distribution, simulation problem type of our case is dynamic, discrete event and stochastic, respectively. After adapting the conceptual model and collecting necessary data, the simulation model was implemented by Arena software. In next section, we will check the model in details.

### The components of Arena model

Arena models include some components such as Entities, Resources, Queues, Variables, and so on. Entities are something like raw materials in manufacturing system or customers of a bank that can be moved and changed in a system. Resources include workforces, machines, warehouses, etc. Queues occur when entities cannot move on, like raw material that may be buffered in a place of production line and waiting for next operation. The components of the Arena model are provided in Table 1.

**Table 1:** The components of the model.

Components	
Entity	Raw oil
Resources	Bascule, Inspectors, PX 60, SRG, SSG, Dryer, Discoloring storage, Operators of odorlessing section, Operators of partitioning section, Operators of hydrogenation section, Operators of filtering section, Operators of post bleach section, and Serak machine
Queues	Bascule queue, Laboratory 1 queue, PX60 separator queue, SRG separator queue, SSG queue, Dryer queue, Discolor queue, Odorless queue, Partitioning queue, Hydrogenation queue, Serak machine queue, and Post bleach queue
Group Resources	Inspectors, Operators of odorless section, Operators of partitioning section, Operators of hydrogenation, Operators of filtering section, and Operators of post bleach section
Transporters	Oil tanker and Liftruck
Conveyers	Conveyer
Processes	Weighting, Laboratory test, PX60 separator, First washing, Second washing, Drying, Discoloring storages, Odorless, Packaging storages, Serak machine, Packaging, Hydrogenation, Filtering, Post bleach, Cold Storage, and Vacating
Scheduling	Regarding to production line that works in three parts of 8 h of a day, scheduling should be done in every section that operators work. Operators who were scheduled are the current operators in group resources
Inputs	Entrance of raw oil
Outputs	Returning oil, Fatty acid storages, Soup storages, First of neutral line, Solid oil storages, and liquid oil storages
Variables	Total oil in process and maximum of oil entrance

### Arena simulation implementation

To implement the Arena model of a production line, we need to know production processes in the shape of a process flowchart. In the previous sections, we discussed the processes of the production line and illustrated the conceptual model of our case. We considered the conceptual model and set our input data in the Arena software. The accuracy of input data in modeling is an important tip in as much as unsuitable input data leads to unsuitable output data. In this research, we had some limitations in data gathering because using time study was not allowed by the production manager. Therefore, we collected the times of the processes (transaction time of the resources) and other data we needed by interviewing the production manager. In most of the processes, the times were in the shape of minimum and maximum. Thus, we allocated uniform probability distribution. However, in some cases, there were exact and constant times. Moreover, one process time was in the shape of minimum, maximum and mod. So, we fit a triangular probability distribution. The transaction times of the resources are provided in Table 2.

**Table 2:** Transaction time of resources.

Processes	Number of operators in per shift	Constant (min)	Minimum (min)	Maximum (min)	Mod (min)
Entrance raw oil (quintuplet batch size)	–	–	300	420	–
Bascule	–	–	6	8	–
Laboratory test 1	1	3	–	–	–
Vacating oil in storages	–	–	35	210	–
PX60 separator	–	–	60	65	–
SRG separator	–	–	60	65	–
SSG separator	–	–	60	65	–
Dryer	–	–	3	4	–
Bleaching	–	–	20	30	–
Storages 1	–	–	1	1440	120
Odorlessing 1 and 2	1	240	–	–	–
Packaging storages	–	–	30	600	–
Serak machine	–	–	2	3	–
Packaging 1	6	0.5	–	–	–
Filtering	2	–	30	60	–
Post bleach	1	–	70	80	–
Storages 2	–	–	30	60	–
Packaging 2	6	–	10	60	–
Cold storage	–	–	720	900	–

**Figure 3:**
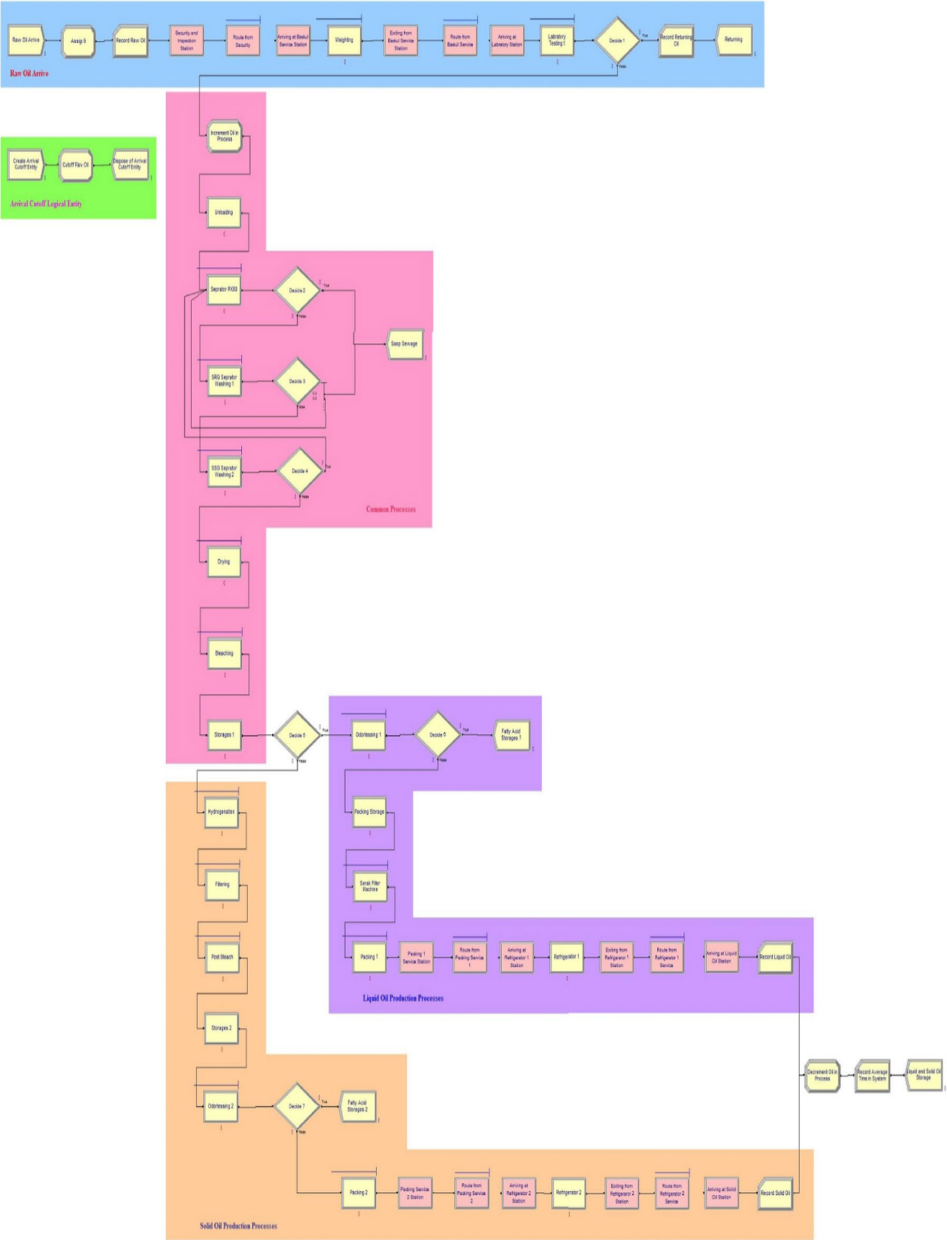
The Arena model.

We use Arena simulation package 13.5 to simulate our model. The designed model by Arena software is illustrated in Fig. 3.

**Figure 4:**
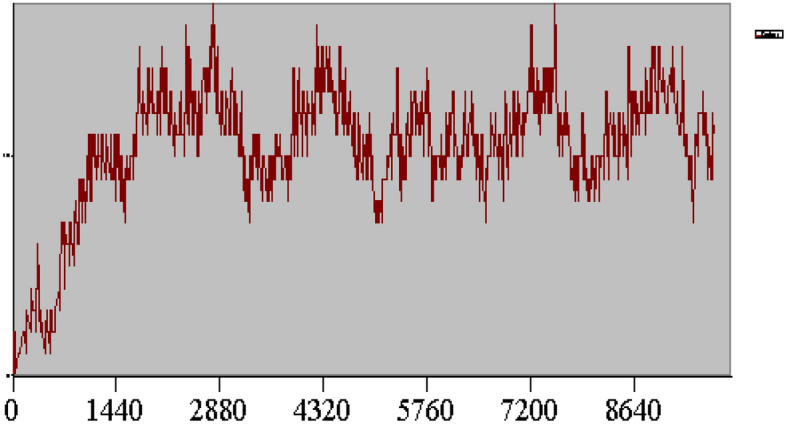
Determining warm up period chart.

The arena model will be run until Terminating Condition is true i.e. the work-in-process (WIP) is equal to 0 and the entrance time of the entity (TNOW) is exceeded over the day (i.e. 86,400 s). The warm-up period is the length of time that the model reaches a stable state. Regarding to Fig. 4, warm-up period was considered approximately 3 days. We can see that after 4320 min, the Arena model reaches a stable state. Thus, warm-up period in terms of days will be in 3 days.

Replication Length means the duration of simulation running time. When we set replication length to infinity, Terminating Condition will be applied. And, replication number means the times the Arena model should be run. Because the production system of the case is continuous, the replication length initialized infinity with 24 h per day. The replication number of running the model will be calculated from the following formula:

1 $$n= {n}_{0}\frac{{h}_{0}^{2}}{{h}^{2}}={n}_{0}\frac{{h}_{0}^{2}}{{\left(0.05* \overline{x}\right)}^{2}}=1* \frac{{\left(0.998691030\right)}^{2}}{{\left(0.05*4.1304\right)}^{2}} \cong 23,$$

where $n$ shows the total replication number of running the model, ${n}_{0}$ is the first time the model is run by replication number 1, ${h}_{0}$ is the initial half width obtained from running the model with replication number 1 from one of the statistical accumulators, and $\overline{x}$ is the average waiting time of the same statistical accumulator^1^.

## Verification and validation

A model (physical, analytical and simulation) is a simple form of the system and contains characteristics of the system. Therefore, we cannot expect that a model can predict the performance of the system exactly. Hence, the main objective of modeling is to make an acceptable and correct representation. To evaluate the correctness of a model (often called Goodness of Fit Test), two components, verification and validation, should be perused^23^.

### Verification

Verification is related to evaluating the integrity of a model that is considered for a simulation problem^23^. In this study, the Arena model will be verified in two ways. First, the conceptual model of our problem was extracted by using OPC and by interviewing the production manager. Second, an animation model of the arena model was designed. After running the model, it was found that the animation model conforms to the real model.

### Validation

Validation tries to evaluate how the hypotheses of the model are realistic by using performance indices. In addition, it depends completely on the validity of the input data^23^. In this study, we did not have permission to calculate the time of the processes by chronometer. Therefore, we interviewed the production manager to confirm our input data. The most important way to validate a model is to represent that the outputs of the simulated model accord with the actual system. In this paper, the output data of the Arena model nearly conformed to the real outputs of the company. As an example, according to historical data, total production is 36 tons per day, and the output of our model indicates that total production is 35.5 tons per day. Thus, our input data is valid.

## Discussion

**Figure 5:**
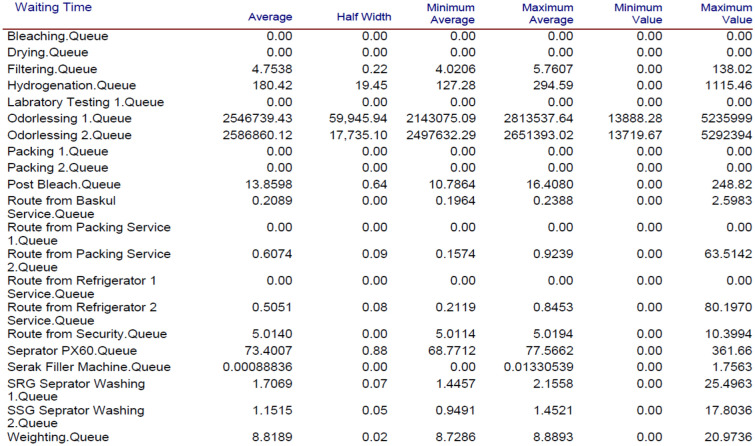
Waiting times before adding changes.

**Figure 6:**
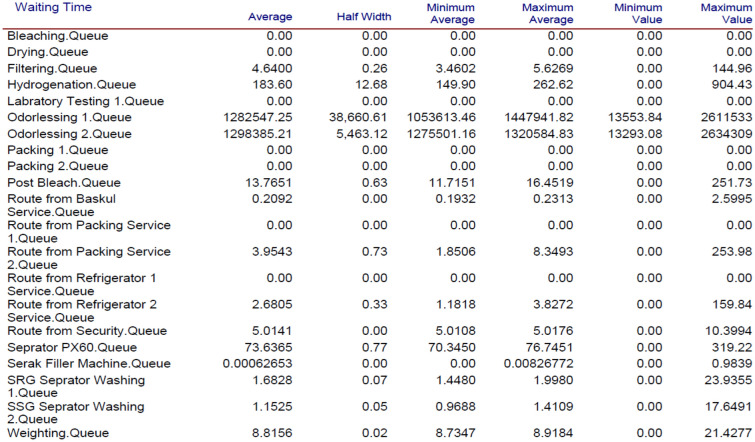
Waiting times after inserting new operators.

In this paper, the conceptual model of the production line of our case was implemented. After that, our necessary data such as transaction time of resources were collected, a simulation model was created, and outputs of the model were obtained. Drawing on the outputs, the average waiting times in queues of the Odorlessing 1 and Odorlessing 2 processes are 4.8 and 4.9 years, respectively, in comparison with the total oil in the system. Therefore, bottlenecks of the system were determined in sections Odorlessing 1 and 2 due to their much waiting time value, and the main reason is the lack of operators in these workstations. Thus, we recommended that some operators be added to these sections. First, we added one operator, then two, three and four more. Increasing new operators can be done until marginal profit of unit end-item will be kept. Therefore, we consider adding three operators to Odorlessing workstations. After this change, we ran our new Arena model by considering new operators. The outputs of the average waiting time in queues of Odorlessing 1 and 2 decreased nearly 50% (2.44 and 2.47 years in comparison with the total oil in system). Therefore, if new workforces are added to the Odorlessing 1 and 2 section, the waiting time in these workstations will decrease. Additionally, because of that, the total production time will be reduced, and the end-item count will be enhanced. As a result of this change, some production costs, such as overhead costs and personnel’s work overtime salaries costs, will be decreased. The results of the Arena model, before applying changes and after adding the operators in Odorlessing 1 and 2 workstations, have been provided in Figs. 5 and 6, repectively.

## Conclusion

This research presented an application of simulation modeling in manufacturing systems by using the Arena simulation software package to evaluate system changes. To evaluate the system of the case today and confirm oncoming projects and enhancements of the system, the model has been implemented. According to the advantages of simulation, the model allows managers to examine production lines without disrupting the current business environment production and to predict the future with confidence. Drawing on the literature, the conceptual model of the processes was obtained in the first step. Next, this process was improved by expanding the model in a graphical environment that allowed the user to establish linkage between the model and the real system.

Any business environment, from customer service to manufacturing to health care, can benefit from simulation. For future studies, additional improvement of our case can be evaluated by developing the provided simulation model. However, it is recommended that the model include other departments of the case. Moreover, it is suggested to model an inventory system for raw materials to determine the reorder point and optimal order quantity with an objective function that minimizes the total cost of the inventory system by using the OptQuest optimizer, an application of the Arena simulation software package.

## Data Availability

All data generated or analyzed during this study are included in this published article.
